# Reproduction predicts shorter telomeres and epigenetic age acceleration among young adult women

**DOI:** 10.1038/s41598-018-29486-4

**Published:** 2018-07-23

**Authors:** Calen P. Ryan, M. Geoffrey Hayes, Nanette R. Lee, Thomas W. McDade, Meaghan J. Jones, Michael S. Kobor, Christopher W. Kuzawa, Dan T. A. Eisenberg

**Affiliations:** 1 Department of Anthropology, Northwestern University, Evanston, IL 60208 USA; 2 Division of Endocrinology, Metabolism and Molecular Medicine, Department of Medicine, Northwestern University, Feinberg School of Medicine, Chicago, IL 60611 USA; 3 Center for Genetic Medicine, Northwestern University Feinberg School of Medicine, Chicago, IL 60611 USA; 4 Office of Population Studies Foundation Inc., Cebu City, Philippines; 5 Department of Anthropology, Sociology, and History, University of San Carlos, Cebu City, Philippines; 6 Institute for Policy Research, Northwestern University, Evanston, IL 60208 USA; 7 Child and Brain Development Program, Canadian Institute for Advanced Research, Toronto, ON M5G 1Z8 Canada; 8 BC Children’s Hospital Research Institute, University of British Columbia, Vancouver, BC V5Z 4H4 Canada; 9 Department of Anthropology, University of Washington, Seattle, WA 98195 USA; 10 Center for Studies in Demography and Ecology, University of Washington, Seattle, WA 98195 USA

## Abstract

Evolutionary theory predicts that reproduction entails costs that detract from somatic maintenance, accelerating biological aging. Despite support from studies in human and non-human animals, mechanisms linking ‘costs of reproduction’ (CoR) to aging are poorly understood. Human pregnancy is characterized by major alterations in metabolic regulation, oxidative stress, and immune cell proliferation. We hypothesized that these adaptations could accelerate blood-derived cellular aging. To test this hypothesis, we examined gravidity in relation to telomere length (TL, n = 821) and DNA-methylation age (DNAmAge, n = 397) in a cohort of young (20–22 year-old) Filipino women. Age-corrected TL and accelerated DNAmAge both predict age-related morbidity and mortality, and provide markers of mitotic and non-mitotic cellular aging, respectively. Consistent with theoretical predictions, TL decreased (p = 0.031) and DNAmAge increased (p = 0.007) with gravidity, a relationship that was not contingent upon resource availability. Neither biomarker was associated with subsequent fertility (both p > 0.3), broadly consistent with a causal effect of gravidity on cellular aging. Our findings provide evidence that reproduction in women carries costs in the form of accelerated aging through two independent cellular pathways.

## Introduction

Evolutionary theory predicts that energy expenditure in the form of reproductive effort comes at the expense of somatic maintenance and lifespan^1^. Because resources are finite and selection favors early life fecundity over late life functional integrity^2^, reductions in somatic maintenance driven by the ‘costs of reproduction’ (CoR) are expected to accelerate senescence and functional decline and increase mortality risk^3,4^. When extrinsic mortality is high or resources are limited or unpredictable, selection will favor future discounting and a shift towards ‘faster’ life-history strategies^1,5^. While potentially adaptive from an evolutionary point-of-view, investing less into growth and maintenance and more into reproduction early in life could compound tradeoffs between reproduction and longevity and thereby accelerate senescence^2,6,7^.

CoR have been demonstrated in animal models, whereby reproduction hastens senescence^8,9^; conversely, selection for late life fecundity results in lifespan extension^10,11^. In humans, CoR has been predominantly studied through the use of historical datasets, which show that increased reproductive effort is often associated with a shortening of lifespan^12–16^, but see^17^ and that these costs are exacerbated when resources are limited^18–20^. However, most studies of CoR in humans are restricted to modeling mortality as the sole outcome, and are therefore unable to address the underlying biological processes through which CoR might translate into senescence and functional decline.

Among women, CoR likely accumulate predominantly during lactation and pregnancy^21,22^. Lactation is energetically taxing, while the highly invasive hemochorial placentation of human pregnancy places substantial physiological and immunological demands on the female body^23–25^. At the cellular level, pregnancy-induced senescence may be mediated through mitotic or non-mitotic pathways, or both. Mitotic - or replicative - cellular aging can be measured using telomere length (TL). Telomeres are non-coding DNA sequences that cap chromosomes, and are required for cell division and survival^26,27^. Telomere length shortens with cell division and chronological age, placing a limit on the number of cell divisions^28–30^. At a critical threshold, TL attrition leads to the exhaustion of a cell’s proliferative potential, a process referred to as ‘cellular senescence’^31,32^. Shorter TL controlling for age in turn predicts higher morbidity and mortality rates^33–36^.

Pregnancy may also affect cellular aging through pathways operating independently from TL. A powerful emerging marker of non-mitotic cellular aging is epigenetic age (DNAmAge)^37,38^. DNAmAge in human^39^ and non-human genomes^40–42^ is calculated from methylation at a species-specific subset of cytosine-guanine dyads (CpGs), and is strongly correlated with chronological age^38,43^. Independent of a host of associated risk factors in humans, accelerated DNAmAge relative to chronological age is associated with elevated risks for morbidity and mortality^44–46^. Vital to capitalizing on epigenetic age as a marker of non-mitotic cellular aging, accelerated DNAmAge predicts senescence and mortality independently of TL in living humans^47,48^, and independently of both TL and the DNA damage response *in vitro*^37,39^.

Human pregnancy could generate costs to female health and lifespan by shortening TL (mitotic age), accelerating DNAmAge (non-mitotic age), or both. During pregnancy, blood cells proliferate to compensate for fluid volume expansion^49,50^, and women experience a shift towards innate immunity and an increased sensitivity to infection^51–54^. Data from cell culture, rodent based experiments, and clinical studies show that inflammation and infection increase cell proliferation and DNA damage, both expected to accelerate the pace of telomere shortening^55–62^. Accelerated DNAmAge relative to chronological age has been observed in other pro-inflammatory contexts^63,64^, and with menopause^65^, an important physiological and life-history transition in human females. DNAmAge acceleration arising from menopause, whether naturally-occurring or surgically-induced is attenuated by hormone therapy^65^, suggesting that physiological and hormonal changes like those accompanying pregnancy could have effects on DNAmAge. While recent studies examining TL or DNA damage and pregnancy have yielded mixed results^66–69^, none have attempted to test for CoR in humans using mitotic and non-mitotic measures of cellular aging simultaneously.

Here, we test for human CoR using mitotic (TL) and non-mitotic (Horvath’s DNAmAge^39^) measures of cellular aging. We test three inter-related hypotheses in a relatively young cohort (age 20–22) of women in the Philippines. First, we ask whether pregnancy history increases mitotic or non-mitotic measures of cellular aging, or both (H1). We consider whether any associations between reproductive history and cell aging are stronger among women of lower socioeconomic status, for whom resource constraints are expected to be highest (H2). Finally, we evaluate the potential for reverse causation by examining the effect of both TL and DNAmAge on the number of pregnancies over the subsequent 4 years (H3).

## Results

The relatively young women in our sample (21.7 ± 0.4 years old) displayed a range of reproductive histories. While women who had never been pregnant formed the largest group (n = 507; 61.7%), women having experienced one (n = 174; 21.2%), two (n = 102; 12.4%), and three (n = 28; 3.4%) pregnancies were also well represented. A small subset of women had experienced four (n = 7; 0.8%) or five (n = 3; 0.4%) pregnancies. Although the women in our sample fell into a relatively narrow age range, age-adjusted measures of DNAmAge and TL were themselves uncorrelated (p = 0.64; n = 396), consistent with their independent roles in cellular aging.

### Reproductive History and Cellular Aging

**Figure 1:**
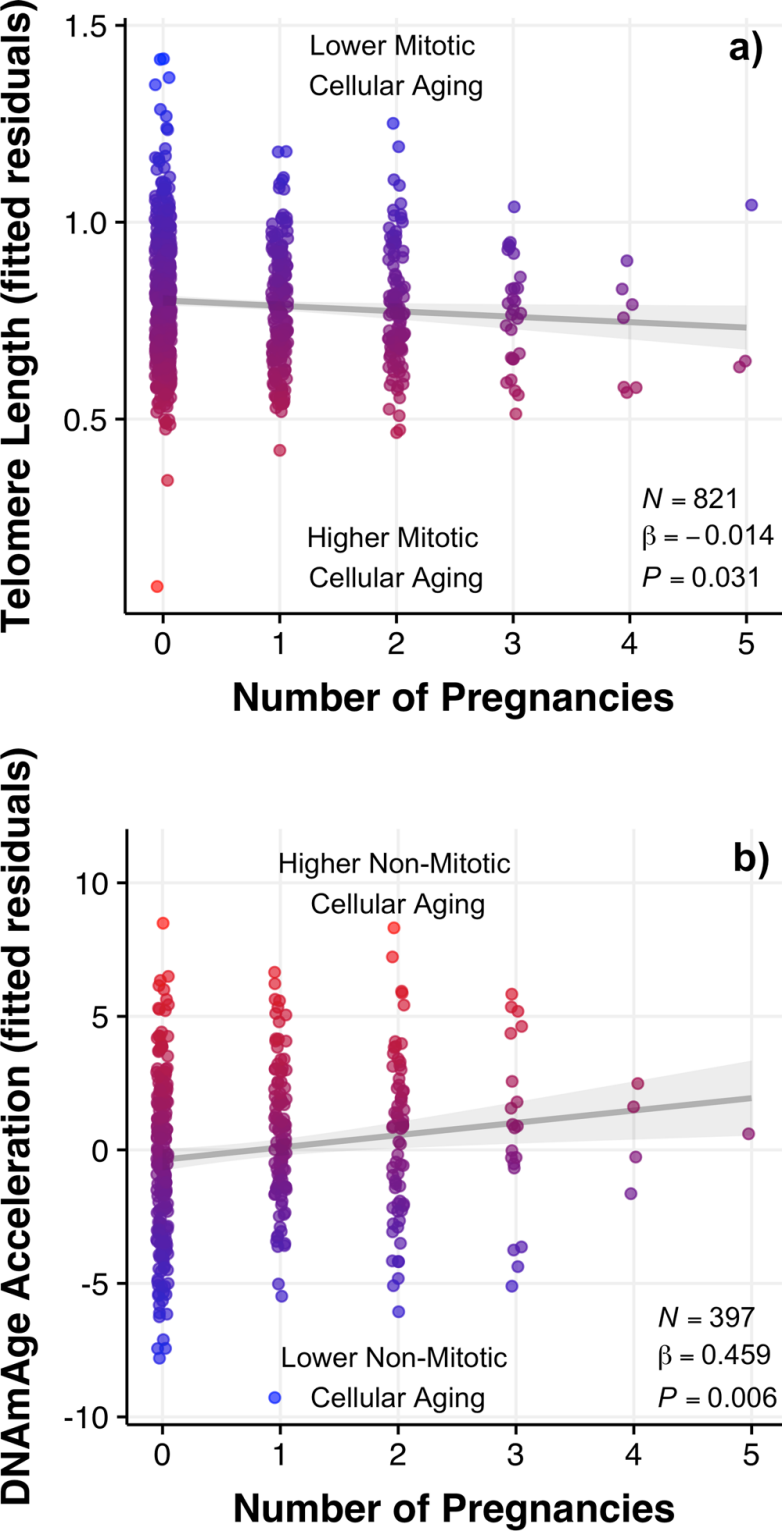
Relationship between mitotic (TL) and non-mitotic (DNAmAge acceleration) measures of cellular aging and reproductive history (number of pregnancies) in young women. (**a**) Residualized TL for all variables in Table 2, Model 3, and statistics from same model. (**b**) Residualized DNAmAge for all variables in Table 2, Model 7, and statistics from same model. Graphs are labeled and dots are colored by relative aging for each marker (blue, low; red, high) and best fit lines are drawn with 95% CI of beta value.

TL decreased and DNAmAge acceleration increased with the number of pregnancies in a woman’s reproductive history (Fig. 1 and Table 1). The relationship between gravidity and both measures were also relatively robust - in nested models controlling for a range of potential confounders, effects sizes for pregnancy number remained stable or increased (Table 1). Each additional pregnancy was associated with the equivalent of 0.34–3.67 years of telomere aging, and 0.29–0.63 years of DNAmAge acceleration (calculations in Supplementary Notes).

**Table 1:** Regression models linking number of pregnancies to telomere length (models 1–4) and DNAmAge (models 5–8).

	Telomere Length				DNAmAge			
	(1)	(2)^†^	(3)^†^	(4)^†^	(5)	(6)^†^	(7)^†^	(8)^†^
Age	−0.047	−0.029	−0.028	−0.029	0.485	0.667	0.656	0.645
*p*-values	0.003**	0.071^+^	0.073^+^	0.068^+^	0.293	0.157	0.158	0.165
No.Pregnancies	−0.014	−0.013	−0.014	−0.016	0.363	0.326	0.459	0.510
*p*-values	0.025*	0.039*	0.031*	0.020*	0.026*	0.049*	0.007**	0.005**
SES		−0.006	−0.006	−0.004		−0.180	−0.214	−0.291
*p*-values		0.143	0.161	0.395		0.146	0.081^+^	0.055^+^
Currently Pregnant (Y)			0.011	0.011			−1.472	−1.460
*p*-values			0.534	0.540			0.001**	0.001**
No. Pregnancies × SES				−0.004				0.106
*p*-values				0.362				0.385
Intercept	1.826	1.337	1.332	1.343	14.818	10.319	10.611	10.850
Observations	821	821	821	821	397	397	397	397
Adjusted R ^2^	0.015	0.063	0.062	0.062	0.011	0.041	0.067	0.067

^†^Marked models include controls for top 10 principal components of genetic variation and average urbanicity score (complete results in Supplementary Table S1). *Note:* ^+^*p* < 0.1; *p < 0.05; **p < 0.01.

### Cellular Aging and Subsequent Parity

We also tested for reverse causation by examining the associations of TL and DNAmAge with future reproduction. Neither measure of cellular aging at the time of measurement (2005) predicted the number of pregnancies over the subsequent four years (2005–2009), whether or not we controlled for baseline gravidity in 2005 (Table 2).

**Table 2:** Relationship between telomere length (TL) and epigenetic age (DNAmAge acceleration) measured in 2005 and parity over the subsequent four years (2005–2009). Models with and without adjustment for baseline gravidity in 2005.

	Parity 2005–2009			
	Telomere Length		DNAmAge	
	Unadjusted for Gravidity in 2005	Adjusted for Gravidity in 2005	Unadjusted for Gravidity in 2005	Adjusted fo rGravidity in 2005
Measurement time bt. 2005–2009 (Days)	−0.003	−0.003	−0.002	−0.002
*p*-values	0.005**	0.011*	0.058^+^	0.068^+^
Parity in 2005		0.252		0.123
*p*-values	0.000**		0.016*	
Age Adjusted Telomere Length in 2005	0.041	0.155		
*p*-values	0.885	0.588		
Age Adjusted DNAmAge in 2005			−0.011	−0.016
*p*-values		0.484	0.325	
Intercept	4.313	3.719	3.461	3.267
Observations	738	738	397	397
Log Likelihood	−832.737	−814.371	−485.277	−482.434
Akaike Inf. Crit.	1,671.474	1,636.742	976.553	972.867

*Note:* ^+^*p* < 0.1; *p < 0.05; **p < 0.01.

## Discussion

TL and DNAmAge, measures of mitotic and non-mitotic cellular aging, respectively, were both associated with reproductive history in our sample of young women. The relationship between gravidity and cellular aging was relatively robust to a number of potential confounders, and did not appear to be mediated by socioeconomic status, a measure of resource availability. Moreover, neither measure was associated with gravidity over the subsequent 4 years, consistent with a causal effect of the number of pregnancy on cellular aging.

Although consistent with theoretical predictions and non-human animal work, this is the first study to our knowledge to examine CoR using both mitotic and non-mitotic measures of cellular aging. Gravidity predicted age-related changes in both TL and DNAmAge in our study, yet several recent studies of CoR in women using TL alone did not find the predicted relationship. The first, conducted among 75 Guatamalan Maya women, reported a positive association between TL and number of surviving offspring over a 13-year period^66^. TL in that study was determined using a combination of saliva- and buccal-derived DNA samples, which unfortunately have not been consistently associated with chronological age^70–72^. Furthermore, two separate measures of TL in that study were uncorrelated within individuals between the two timepoints, making comparisons between these findings and our own blood-derived TL findings difficult.

Contrasting with our findings, a study among 620 participants of the US-based CARDIA study did not find evidence for any relationship between parity and TL^69^. Why this study found no evidence for an effect of parity on TL while our findings support CoR is unclear, but could relate to pronounced differences in the age ranges and socio-ecological conditions in the two populations. Notably, markers of oxidative stress appear to be affected by parity in some socio-ecological contexts but not others^67,68^. Furthermore, TL attrition occurs more rapidly at younger ages^73^, suggesting that any impacts of reproduction on TL shortening could be most pronounced among young women, especially if reproduction begins in adolescence and overlaps with late stages of the mother’s own somatic growth^1,74^. Whether or not the relationship between TL and DNAmAge will persist, or if women with accelerated cellular aging will ‘recover’ and return to more age-typical levels remains an open question.

We found evidence for CoR using both TL and DNAmAge, yet these two measures of cellular aging appear to reflect different biological pathways linking reproductive effort with senescence. Congruent with this interpretation is the observation that TL and DNAmAge measured in the same individuals have been independently associated with aging and mortality in prior studies^48,75^, and capture distinct dimensions of cellular aging^37,38,76^. Accordingly, TL and DNAmAge acceleration were not associated with each other in this study. Accelerated TL attrition - a measure of ‘mitotic age’ that is modified directly by cellular division - could stem from factors that modify cellular proliferation rates, such as the elevated inflammation, blood cell production, and cell-turnover rates that characterize pregnancy in this and other samples^25,77^.

In contrast to TL, Horvath’s DNAmAge is not considered a marker of mitotic age. *In vitro* DNAmAge is associated with cell passage number, but only in conjunction with the expression of the Telomerase Reverse Transcriptase (TERT) gene^76^, and DNAmAge tracks chronological age even in immortal, non-dividing, and non-proliferative tissues and cells^39^. Although the biological significance of DNAmAge is unknown, it is hypothesized to reflect the integrity of an epigenetic maintenance system, itself responsible for maintaining dynamic regulatory stability within cells^39^. In light of the hypotheses about the functional underpinnings of DNAmAge, our findings are consistent with the prediction that reproduction comes at a cost of ‘maintenance’ - in this case at the scale of cellular regulatory integrity. Exactly how gravidity might lead to DNAmAge acceleration is unclear, but tradeoffs between protein homeostasis and epigenetic control arising from immune activation or the buffering of oxidative stress are plausible pathways^78–81^. Indeed, cumulative changes in immune cell composition during pregnancy likely contribute to DNAmAge acceleration with gravidity, although the measure of DNAmAge used here is remarkably robust across tissue types^39^. Nevertheless, the fact that the functionally-distinct measures of TL and DNAmAge show similar associations with gravidity provides strong support for our prediction that reproduction accelerates cellular aging and organismal senescence, at least among the young adult women represented by our sample.

Contrary to our prediction that the costs of reproduction would be greatest among individuals with limited resources^18–20^, we found no evidence for an interaction between gravidity and SES in models predicting either TL or DNAmAge acceleration. While women in low SES conditions in our study very likely experience constraints in energy or nutrient availability, it is still unclear to what extent our measure of SES adequately captures limitations in the resources most relevant to CoR. Given the relatively young age of the participants, however, it is possible that the moderating effect of resource limitation will emerge at more advanced ages. SES in this population may also index factors other than resource availability that contribute to accelerated aging, such as less healthful diets or decreases in physical activity. This does not appear to be a major confounding factor, however, as neither TL or DNAmAge were significantly associated with SES in our models.

Importantly, neither measure of cellular aging obtained in 2005 predicted parity over the subsequent 4 years (2005–2009). This suggests that the women in our study are not altering their reproductive output based on their future prospects of health and survival, nor in response to separate physiological or environmental factors also responsible for accelerating cellular aging. This runs counter to a life-history framework whereby ‘pace-of-living’ as captured by TL and DNAmAge is itself predictive of future fecundity^5,82^.

Intriguingly, currently pregnant women exhibited significantly ‘younger’ DNAmAge. This finding could reflect the suite of immunological and physiological shifts that occur during pregnancy, including changes in immune cell composition and elevated estrogen levels. At least in some contexts, estrogen can lower oxidative stress^83^, and elevated estrogen is protective for both TL and DNAmAge^65,84^. Pregnancy status and accompanying changes in cell composition may therefore be an important confounder to include in future studies investigating the costs of reproduction in women.

Our findings should be considered in the context of several limitations. First, while we attempt to control for socio-ecological factors that could affect both gravidity and our markers of cellular aging, residual confounding arising from differences in health and/or resources remains a possibility. Although the effects were modest, confounding could help explain the slight decrease in effect size of gravidity after adjusting for SES in models 2 and 6. Future studies employing longitudinal measures of TL and DNAmAge acceleration would minimize the potential effects of such confounders^85^, while modeling lactation and other indices for reproductive effort will be necessary for a more complete estimate of the CoR^86,87^. Finally, the women in this study all fall within a relatively narrow age range in young adulthood (20–22 years old). Because both TL and DNA-methylation change more rapidly early in adulthood^39,73^, it is possible that both measures are particularly sensitive to reproduction at this time. This leaves open the possibility that the relationship between gravidity and cellular aging is transient - and measurements of TL and DNAmAge later in life will prove important for resolving this question.

In sum, our study suggests that gravidity predicts shorter telomeres and epigenetic age acceleration, measures of mitotic and non-mitotic aging, respectively, among the young women in our sample. The consistency in relationships between gravidity and aging in two distinct pathways—one reflecting cellular turnover, and the second a putative marker of epigenomic regulation—support a cost of reproduction from pregnancy in humans.

## Methods

### Data collection

Data came from the Cebu Longitudinal Health and Nutrition Survey (CLHNS), a birth cohort study in Metropolitan Cebu, Philippines that began with enrollment of 3,327 pregnant mothers in 1983–1984^88^. Longitudinal data are available for download at: https://dataverse.unc.edu/dataverse/cebu. In 2005 blood samples from overnight fasted subjects were collected into EDTA-coated vacutainer tubes. Automated and manual DNA extraction (Puregene, Gentra) was conducted on blood samples. Informed consent was obtained from all participants and data collection was conducted with approval and in accordance with the Institutional Review Boards of the University of North Carolina at Chapel Hill and Northwestern University.

### Telomere length

TLs were measured using a modified form of the monochrome multiplex quantitative polymerase chain reaction assay that was externally validated. Details of the protocol and external validity can be found in^89^ and since the coefficient of variation has recently been recognized to be an invalid statistic to assess TL measurement reliability^90,91^, intraclass correlation coefficient statistics of measurement error can be found in^92^.

### Epigenetic age (DNAmAge)

160 ng of sodium bisulfite converted DNA (Zymo AZDNA methylation kit, Zymo Research, Irvine, CA, USA) was applied to the Illumina HumanMethylation450 Bead Chip using manufacturer’s standard conditions. Standard methods for background subtraction and color correction were carried out using default parameters in Illumina Genome Studio and exported into R for further analyses. Quality control involved first confirming participant sex and replicate status. This was followed by quantile normalization using lumi^93^ on all probes including SNP-associated and XY multiple binding probes. To maximize the number of sites available for the epigenetic age calculator, probes with detection p-values above 0.01 were called NA for poor performing samples only, and were otherwise retained. Horvath’s DNAmAge was calculated using an online calculator (http://labs.genetics.ucla.edu/horvath/dnamage/), designed to be generally robust to cell-type differences associated with age^39^. Background-corrected beta values were pre-processed using the calculator’s internal normalization algorithms.

### Socioeconomic status (SES)

SES is measured as a combination of income, education, and assets. Participants reported their annual income from all sources, including in-kind services, and the sale of livestock or other products by household members during the prior year, which were summed to determine total household income. Incomes were deflated to 1983 levels, and log-transformed. Maternal education (in years) was also reported. Participants also reported on nine assets (coded 0, 1) that were selected to capture population-relevant aspects of social class, including electricity, televisions, refrigerators, air conditioners, tape recorder, electric fans, jeepneys, cars, and their residence. In addition, house construction type (i.e., light, mixed, permanent structure) was coded as 0, 1, and 2, respectively. Thus, asset scores ranged from 0 to 11. A principal components analysis was run on log income and assets at birth (1983) and at sample collection (2005) along with maternal education in Stata (v. 14.1). The first component of variation accounted for 49% of the variation and individual scores for this component of variation were used as our measure of SES.

### Statistical methods

The key predictor variable was gravidity (the number of pregnancies including stillbirths, miscarriages and live births, but not current pregnancies) the respondent reported having had in 2005 (at the time of blood sampling). Control variables included chronological age in 2005 (the time of blood collection), the measure of socioeconomic status (SES) described above, average urbanicity score between 1983 and 2005^94^, and whether the respondent was pregnant at the time of blood collection. Pregnancy status was reported at the time of sampling, and through back-calculation based on parturition within 9 months of the original interview (maternal and infant measures are recorded with each pregnancy as part of ongoing tracking process). DNAmAge acceleration refers to DNAmAge residualized on chronological age. Principal components (PCs) of genome-wide genetic variation were included to control for potential population genetic structure. The derivation of these principal components has been described previously^95–97^. As in previous analyses^92,98^, the bivariate association between the first ten principal components and TL were tested. The top principal components up to and including the last one showing a significant bivariate association with TL (10 total) were retained as control variables, with the same 10 principal components used for DNAmAge models.

Linear regression was used for analyses predicting TL and DNAmAge (both normally distributed continuous outcome variables), while generalized linear models with a Poisson family and log-link were used to test for reverse association - that TL/DNAmAge would predict gravidity (a discrete integer) over the subsequent 4 years. The negative effect of time between 2005–2009 surveys and number of pregnancies during this time is an artifact tied to household visit schedules and urbanicity (less urban participants tend to have more pregnancies, and were visited later in the data collection wave). All models were two-tailed with *α* = 0.05 and were followed by standard model diagnostics^99^. For all linear regressions, the absence of collinearity in predictor variables was confirmed with variance inflation factors (VIFs) for all models falling below 1.1, while Poisson GLMs showed no signs of under- or over-dispersion^100^. Despite the large number of nulliparous women and relatively small number of women with 3 or more pregnancies, all model assumptions were met, and there was no evidence of heteroscedasticity, outliers, or high leverage data points confounding our analyses. All analyses were run in R^101^ with ggplot2^102^ and stargazer^103^ for figures and tables.

## Electronic supplementary material

Supplementary Information

## References

[1] Stearns SC (1992). The Evolution of Life Histories.

[2] Williams, G. C. Pleiotropy, Natural Selection, and the Evolution of Senescence. *Evol*. 398–411 (1957).

[3] Kirkwood TBL (1977). Evolution of ageing. Nat..

[4] Harshman, L. G. & Zera, A. J. The cost of reproduction: the devil in the details. *Trends Ecol*. *& Evol*. **22**, 80–86, http://www.sciencedirect.com/science/article/pii/S0169534706003417, 10.1016/j.tree.2006.10.008 (2007). 10.1016/j.tree.2006.10.008 17056152

[5] Nettle D (2010). Dying young and living fast: variation in life history across English neighborhoods. Behav. Ecol..

[6] Kuzawa CW (2007). Developmental origins of life history: Growth, productivity, and reproduction. Am. J. Hum. Biol..

[7] Jasienska, G., Bribiescas, R. G., Furberg, A.-S., Helle, S. & Núñez-de la Mora, A. Human reproduction and health: an evolutionary perspective. *The Lancet* **390**, 510–520, http://www.sciencedirect.com/science/article/pii/S0140673617305731 (2017). 10.1016/S0140-6736(17)30573-1 28792413

[8] Maynard Smith, J. The Effects of Temperature and of Egg-Laying on the Longevity of Drosophila Subobscura. *J*. *Exp*. *Biol*. **35**, 832–842, http://jeb.biologists.org/content/35/4/832 (1958).

[9] Reznick D (1985). Costs of reproduction: an evaluation of the empirical evidence. Oikos.

[10] Curtsinger JW (1995). Genetic variation and aging. Annu. Rev Genet..

[11] Rose MR (2002). Evolution of late-life mortality in drosophila melanogaster. Evol..

[12] Westendorp RGJ, Kirkwood TBL (1998). Human longevity at the cost of reproductive success. Nat..

[13] Doblhammer, G. & Oeppen, J. Reproduction and longevity among the British peerage: the effect of frailty and health selection. *Proc*. *Royal Soc*. *Lond*. *B*: *Biol*. *Sci*. **270**, 1541–1547, http://rspb.royalsocietypublishing.org.turing.library.northwestern.edu/content/270/1524/1541, 10.1098/rspb.2003.2400 (2003). 10.1098/rspb.2003.2400 PMC1691410 12908973

[14] Penn, D. J. & Smith, K. R. Differential fitness costs of reproduction between the sexes. *Proc*. *Natl*. *Acad*. *Sci*. **104**, 553–558, http://www.pnas.org.turing.library.northwestern.edu/content/104/2/553, 10.1073/pnas.0609301103 (2007). 10.1073/pnas.0609301103 PMC1766423 17192400

[15] Gagnon A (2009). Is there a trade-off between fertility and longevity? A comparative study of women from three large historical databases accounting for mortality selection. Am. J. Hum. Biol..

[16] Bolund, E., Lummaa, V., Smith, K. R., Hanson, H. A. & Maklakov, A. A. Reduced costs of reproduction in females mediate a shift from a male-biased to a female-biased lifespan in humans. *Sci*. *Reports* **6**, 24672, http://www.nature.com/articles/srep24672, 10.1038/srep24672 (2016). 10.1038/srep24672 PMC4834564 27087670

[17] Le Bourg, E. Does reproduction decrease longevity in human beings? *Ageing Res*. *Rev*. **6**, 141–149, http://www.sciencedirect.com/science/article/pii/S1568163707000207, 10.1016/j.arr.2007.04.002 (2007). 10.1016/j.arr.2007.04.002 17532269

[18] Tracer DP (1991). Fertility-related changes in maternal body composition among the au of Papua New Guinea. Am. J. Phys. Anthropol..

[19] Lycett, J. E., Dunbar, R. I. M. & Voland, E. Longevity and the costs of reproduction in a historical human population. *Proc*. *Royal Soc*. *Lond*. *B*: *Biol*. *Sci*. **267**, 31–35, http://rspb.royalsocietypublishing.org/content/267/1438/31.short (2000). 10.1098/rspb.2000.0962 PMC1690499 10670949

[20] Dribe M (2004). Long-term effects of childbearing on mortality: evidence from pre-industrial Sweden. Popul. Stud..

[21] Speakman J, Kro’l E (2005). Limits to sustained energy intake IX: a review of hypotheses. J. Comp. Physiol. B: Biochem. Syst. Environ. Physiol..

[22] Jasienska G (2009). Reproduction and lifespan: Trade-offs, overall energy budgets, intergenerational costs, and costs neglected by research. Am. J. Hum. Biol..

[23] Ellison, P. T. On fertile ground: A natural history of human reproduction (Harvard University Press, Cambridge, MA, 2009).

[24] Emery Thompson M (2013). Comparative Reproductive Energetics of Human and Nonhuman Primates. Annu. Rev. Anthropol..

[25] Soma-Pillay, P. *et al*. Physiological changes in pregnancy. *Cardiovasc*. *J*. *Afr*. **27**, 89–94, https://www.ncbi.nlm.nih.gov/pmc/articles/PMC4928162, 10.5830/CVJA-2016-021 (2016). 10.5830/CVJA-2016-021 PMC4928162 27213856

[26] Blackburn EH, Gall JG (1978). A tandemly repeated sequence at the termini of the extrachromosomal ribosomal RNA genes in Tetrahymena. J. Mol. Biol..

[27] Meyne J, Ratliff RL, Moyzis RK (1989). Conservation of the human telomere sequence (TTAGGG)n among vertebrates. Proc. Natl. Acad. Sci..

[28] Olovnikov AM (1971). Principle of marginotomy in template synthesis of polynucleotides. Dokl Akad Nauk SSSR.

[29] Harley CB, Futcher AB, Greider CW (1990). Telomeres shorten during ageing of human fibroblasts. Nat..

[30] Richter T, Zglinicki T (2007). A continuous correlation between oxidative stress and telomere shortening in fibroblasts. Exp. Gerontol..

[31] Sidler, C., Kovalchuk, O. & Kovalchuk, I. Epigenetic Regulation of Cellular Senescence and Aging. *Front*. *Genet*. **8**, 10.3389/fgene.2017.00138 (2017). 10.3389/fgene.2017.00138 PMC5622920 29018479

[32] Fulop, T. *et al*. Immunosenescence and Inflamm-Aging As Two Sides of the Same Coin: Friends or Foes? *Front*. *Immunol*. **8**, 10.3389/fimmu.2017.01960 (2018). 10.3389/fimmu.2017.01960 PMC5767595 29375577

[33] Cawthon RM, Smith KR, O’Brien E, Sivatchenko A, Kerber RA (2003). Association between telomere length in blood and mortality in people aged 60 years or older. The Lancet.

[34] Bakaysa SL (2007). Telomere length predicts survival independent of genetic influences. Aging Cell.

[35] Kimura M (2008). Telomere length and mortality: a study of leukocytes in elderly Danish twins. Am J Epidemiol.

[36] Haycock PC (2014). Leucocyte telomere length and risk of cardiovascular disease: systematic review and meta-analysis. BMJ.

[37] Lowe, D., Horvath, S. & Raj, K. Epigenetic clock analyses of cellular senescence and ageing. *Oncotarget* **7**, 8524, https://www.ncbi.nlm.nih.gov/pmc/articles/PMC4890984/ (2016). 10.18632/oncotarget.7383 PMC4890984 26885756

[38] Horvath, S. & Raj, K. DNA methylation-based biomarkers and the epigenetic clock theory of ageing. *Nat*. *Rev*. *Genet*. **19**, 371–384, http://www.nature.com/articles/s41576-018-0004-3, 10.1038/s41576-018-0004-3 (2018). 10.1038/s41576-018-0004-3 29643443

[39] Horvath S (2013). DNA methylation age of human tissues and cell types. Genome Biol..

[40] Stubbs TM (2017). Multi-tissue DNA methylation age predictor in mouse. Genome Biol..

[41] Thompson, M. J., von Holdt, B., Horvath, S. & Pellegrini, M. An epigenetic aging clock for dogs and wolves. *Aging* (*Albany NY*) **9**, 1055–1068, http://www.ncbi.nlm.nih.gov/pmc/articles/PMC5391218, 10.18632/aging.101211 (2017). 10.18632/aging.101211 PMC5391218 28373601

[42] Polanowski, A. M., Robbins, J., Chandler, D. & Jarman, S. N. Epigenetic estimation of age in humpback whales. *Mol*. *Ecol*. *Resour*. **14**, 976–987, https://www.ncbi.nlm.nih.gov/pmc/articles/PMC4314680, 10.1111/1755-0998.12247 (2014). 10.1111/1755-0998.12247 PMC4314680 24606053

[43] Jones MJ, Goodman SJ, Kobor MS (2015). DNA methylation and healthy human aging. Aging Cell.

[44] Marioni, R. E. *et al*. DNA methylation age of blood predicts all-cause mortality in later life. *Genome Biol*. **16**, 25, http://genomebiology.com/2015/16/1/25, 10.1186/s13059-015-0584-6 (2015). 10.1186/s13059-015-0584-6 PMC4350614 25633388

[45] Chen BH (2016). DNA methylation-based measures of biological age: meta-analysis predicting time to death. Aging (Albany NY).

[46] Christiansen, L. *et al*. DNA methylation age is associated with mortality in aAˆ longitudinal Danish twin study. *Aging Cell* **15**, 149–154, http://www.ncbi.nlm.nih.gov/pmc/articles/PMC4717264, 10.1111/acel.12421 (2016). 10.1111/acel.12421 PMC4717264 26594032

[47] Breitling LP (2016). Frailty is associated with the epigenetic clock but not with telomere length in a German cohort. Clin. Epigenetics.

[48] Marioni, R. E. *et al*. The epigenetic clock and telomere length are independently associated with chronological age and mortality. *Int*. *J*. *Epidemiol*. dyw041, http://ije.oxfordjournals.org.turing.library.northwestern.edu/content/early/2016/04/13/ije.dyw041, 10.1093/ije/dyw041 (2016). 10.1093/ije/dyw041 PMC4864882 27075770

[49] Lurie S, Rahamim E, Piper I, Golan A, Sadan O (2008). Total and differential leukocyte counts percentiles in normal pregnancy. Eur. J. Obstet. & Gynecol. Reproductive Biol..

[50] Bauer, K. A. Hematologic changes in pregnancy. *UpToDate* (2014).

[51] Roberts CW, Satoskar A, Alexander J (1996). Sex steroids, pregnancy-associated hormones and immunity to parasitic infection. Parasitol. Today.

[52] Lanciers S, Despinasse B, Mehta DI, Blecker U (1999). Increased susceptibility to Helicobacter pylori infection in pregnancy. Infect Dis Obstet Gynecol.

[53] Gray RH (2005). Increased risk of incident HIV during pregnancy in Rakai, Uganda: a prospective study. The Lancet.

[54] Kraus TA (2012). Characterizing the Pregnancy Immune Phenotype: Results of the Viral Immunity and Pregnancy (VIP) Study. J. Clin. Immunol..

[55] Pommier J-P (1997). Immunosenescence in HIV Pathogenesis. Virol..

[56] Aviv A (2006). Menopause Modifies the Association of Leukocyte Telomere Length with Insulin Resistance and Inflammation. J Clin Endocrinol Metab.

[57] Sampson MJ, Winterbone MS, Hughes JC, Dozio N, Hughes DA (2006). Monocyte telomere shortening and oxidative DNA damage in type 2 diabetes. Diabetes Care.

[58] Carrero JJ (2008). Telomere attrition is associated with inflammation, low fetuin-A levels and high mortality in prevalent haemodialysis patients. J. Intern. Medicine.

[59] Farzaneh-Far R (2010). Telomere length trajectory and its determinants in persons with coronary artery disease: longitudinal findings from the heart and soul study. PLoS One.

[60] O’Donovan A (2011). Cumulative Inflammatory Load Is Associated with Short Leukocyte Telomere Length in the Health, Aging and Body Composition Study. PLoS ONE.

[61] Solorio S (2011). Association Between Telomere Length and C-Reactive Protein and the Development of Coronary Collateral Circulation in Patients with Coronary Artery Disease. Angiol..

[62] Sanders JL (2012). Leukocyte telomere length is associated with noninvasively measured age-related disease: The Cardiovascular Health Study. J Gerontol A Biol Sci Med Sci.

[63] Horvath, S. & Levine, A. J. HIV-1 Infection Accelerates Age According to the Epigenetic Clock. *J*. *Infect*. *Dis*. **212**, 1563–1573, http://jid.oxfordjournals.org/content/212/10/1563, 10.1093/infdis/jiv277 (2015). 10.1093/infdis/jiv277 PMC4621253 25969563

[64] Kananen, L. *et al*. Cytomegalovirus infection accelerates epigenetic aging. *Exp*. *Gerontol*. **72**, 227–229, http://linkinghub.elsevier.com/retrieve/pii/S0531556515300711, 10.1016/j.exger.2015.10.008 (2015). 10.1016/j.exger.2015.10.008 26485162

[65] Levine, M. E. *et al*. Menopause accelerates biological aging. *Proc. Natl. Acad. Sci*. 201604558, 10.1073/pnas.1604558113 (2016). 10.1073/pnas.1604558113 PMC4995944 27457926

[66] Barha CK (2016). Number of Children and Telomere Length in Women: A Prospective, Longitudinal Evaluation. PLOS ONE.

[67] Ziomkiewicz, A., Frumkin, A., Zhang, Y., Sancilio, A. & Bribiescas, R. G. The cost of reproduction in women: Reproductive effort and oxidative stress in premenopausal and postmenopausal American women. *Am. J. Hum. Biol*. 30, n/a–n/a, 10.1002/ajhb.23069 (2018). 10.1002/ajhb.23069 28984395

[68] Ziomkiewicz, A. *et al*. Evidence for the cost of reproduction in humans: high lifetime reproductive effort is associated with greater oxidative stress in post-menopausal women. *PloS one* **11**, e0145753, http://journals.plos.org/plosone/article?id=10.1371/journal.pone.0145753 (2016). 10.1371/journal.pone.0145753 PMC4711894 26761206

[69] Lane-Cordova AD (2017). Gravidity is not associated with telomere length in a biracial cohort of middle-aged women: The Coronary Artery Risk Development in Young Adults (CARDIA) study. PLOS ONE.

[70] O’Callaghan N (2008). Buccal cells: a non-invasive measurement of selenium, zinc and magnesium status, and telomere length. Asia Pac. J. Clin. Nutr.

[71] Thomas, P. *Changes in buccal cytome biomarkers in relation to ageing and Alzheimer*’*s disease*. Ph.D. thesis, University of Adelaide http://hdl.handle.net/2440/56186 (2008).

[72] Goldman, E. A. *et al*. Evaluating minimally invasive sample collection methods for telomere length measurement. *Am*. *J*. *Hum*. *Biol*. e23062–n/a (2017). 10.1002/ajhb.23062 PMC5785450 28949426

[73] Frenck RW, Blackburn EH, Shannon KM (1998). The rate of telomere sequence loss in human leukocytes varies with age. Proc. Natl. Acad. Sci..

[74] Hill K, Kaplan aH (1999). Life History Traits in Humans: Theory and Empirical Studies. Annu. Rev. Anthropol..

[75] Belsky, D. W. *et al*. Eleven Telomere, Epigenetic Clock, and Biomarker-Composite Quantifications of Biological Aging: Do They Measure the Same Thing? *Am*. *J*. *Epidemiol*., 10.1093/aje/kwx346 (2018). 10.1093/aje/kwx346 PMC6248475 29149257

[76] Lu, A. T. *et al*. GWAS of epigenetic aging rates in blood reveals a critical role for TERT. *Nat*. *Commun*. **9**, 387, https://www-nature-com.turing.library.northwestern.edu/articles/s41467-017-02697-5, 10.1038/s41467-017-02697-5 (2018). 10.1038/s41467-017-02697-5 PMC5786029 29374233

[77] Kuzawa CW, Adair LS, Borja J, Mcdade TW (2013). C-reactive protein by pregnancy and lactational status among Filipino young adult women. Am. J. Hum. Biol..

[78] Feder ME, Hofmann GE (1999). Heat-shock proteins, molecular chaperones, and the stress response: evolutionary and ecological physiology. Annu. Rev. Physiol..

[79] Marshall KE, Sinclair BJ (2010). Repeated stress exposure results in a survival-reproduction trade-off in Drosophila melanogaster. Proc. Royal Soc. B: Biol. Sci..

[80] Okada Y, Teramura K, Takahashi KH (2014). Heat shock proteins mediate trade-offs between early-life reproduction and late survival in Drosophila melanogaster. Physiol. Entomol..

[81] Ryan CP, Brownlie JC, Whyard S (2016). Hsp90 and physiological stress are linked to autonomous transposon mobility and heritable genetic change in nematodes. Genome Biol. Evol..

[82] Williams, G. C. *Adaptation and natural selection: a critique of some current evolutionary thought*. Princeton science library OCLC: 833082108 (Princeton Univ. Press, Princeton, NJ, 1966).

[83] Behl, C. *et al*. Neuroprotection against Oxidative Stress by Estrogens: Structure-Activity Relationship. *Mol*. *Phar macol*. **51**, 535–541, http://molpharm.aspetjournals.org/content/51/4/535, 10.1124/mol.51.4.535 (1997). 9106616

[84] Yeap, B. B. *et al*. Epidemiological and Mendelian randomisation studies of dihydrotestosterone and estradiol, and leucocyte telomere length in men. *The J*. *Clin*. *Endocrinol*. *& Metab*. *jc*. 2015–4139 (2016). 10.1210/jc.2015-4139 26789780

[85] Noordwijk, A. J. v. & Jong, G. d. Acquisition and Allocation of Resources: Their Influence on Variation in Life History Tactics. *The Am*. *Nat*. **128**, 137–142, http://www.jstor.org/stable/2461293, ArticleType: research-article/Full publication date: Jul., 1986/Copyright Â© 1986 The University of Chicago Press (1986).

[86] Gurven, M. *et al*. Health costs of reproduction are minimal despite high fertility, mortality and subsistence lifestyle. *Sci*. *Reports* **6**, 30056, http://www.nature.com/articles/srep30056, 10.1038/srep30056 (2016). 10.1038/srep30056 PMC4951795 27436412

[87] Helle, S. Accounting for measurement error in human life history trade-offs using structural equation modeling. *Am*. *J*. *Hum*. *Biol*. e23075 10.1002/ajhb.23075 (2017). 10.1002/ajhb.23075 29130592

[88] Adair, L. S. *et al*. Cohort Profile: The Cebu Longitudinal Health and Nutrition Survey. *Int*. *J*. *Epidemiol*. **40**, 619–625, http://www.ncbi.nlm.nih.gov/pmc/articles/PMC3147061, 10.1093/ije/dyq085 (2011). 10.1093/ije/dyq085 PMC3147061 20507864

[89] Eisenberg DT, Kuzawa CW, Hayes MG (2015). Improving qPCR telomere length assays: Controlling for well position effects increases statistical power. Am J Hum Biol.

[90] Verhulst S (2015). Commentary: The reliability of telomere length measurements. Int J Epidemiol.

[91] Eisenberg DT (2016). Telomere length measurement validity: the coefficient of variation is invalid and cannot be used to compare quantitative polymerase chain reaction and Southern blot telomere length measurement techniques. Int J Epidemiol.

[92] Eisenberg, D. T. A., Borja, J. B., Hayes, M. G. & Kuzawa, C. W. Early life infection, but not breastfeeding, predicts adult blood telomere lengths in the Philippines. *Am*. *J*. *Hum*. *Biol*. **29** (2017). 10.1002/ajhb.22962 PMC5511763 28121388

[93] Du P, Kibbe WA, Lin SM (2008). lumi: a pipeline for processing Illumina microarray. Bioinforma..

[94] Dahly DL, Adair LS (2007). Quantifying the urban environment: A scale measure of urbanicity outperforms the urban-rural dichotomy. Soc. Sci. & Medicine.

[95] Croteau-Chonka DC (2011). Genome-wide association study of anthropometric traits and evidence of interactions withage and study year in Filipino women. Obes. (Silver Spring).

[96] Croteau-Chonka DC (2012). Population-specific coding variant underlies genome-wide association with adiponectin level. Hum Mol Genet..

[97] Wu Y (2012). Genome-wide Association with C-Reactive Protein Levels in CLHNS: Evidence for the CRP and HNF1a Loci and their Interaction with Exposure to a Pathogenic Environment. Inflamm..

[98] Bethancourt, H. J. *et al*. No Association between Blood Telomere Length and Longitudinally-Assessed Diet or Adiposity or Diet in a Young Adult Filipino Population. *Eur*. *J*. *Nutr*. 1–14 (2015). 10.1007/s00394-015-1080-1 PMC4846585 26497538

[99] Fox, J. & Weisberg, S. *car*: *Companion to Applied Regression*, second edition edn, http://socserv.socsci.mcmaster.ca/jfox/Books/Companion (SAGE Publications, Thousand Oaks, CA, 2011).

[100] Kleiber, C. & Zeileis, A. *Applied econometrics with R* (Springer Verlag, 2008).

[101] Team, R. C. D. R: A language and environment for statistical computing, reference index version 3.3.1. *R Foundation for Stat*. *Comput*. *Vienna*, *Austria*. (2016).

[102] Wickham, H. & Chang, W. Package ggplot2 (2013).

[103] Hlavac, M. stargazer: LaTeX code and ASCII text for well-formatted regression and summary statistics tables (2014).

