# Supplementary material for: Reproduction predicts shorter telomeres and epigenetic age acceleration among young adult women
Source: Sci Rep. 2018 Jul 23;8:11100. doi: 10.1038/s41598-018-29486-4 (PMC6056536; doi:10.1038/s41598-018-29486-4)
Supplement: Supplementary file 1 — Supplementary Information [file 41598_2018_29486_MOESM1_ESM.pdf]

Reproduction predicts shorter telomeres and epigenetic age acceleration among young adult women

Calen P. Ryan[a,1*], M. Geoffrey Hayes[b,c,d], Nanette R. Lee[e,f], Thomas W. McDade[a,g,h], Meaghan J. Jones[i], Michael S. Kobor[h,i], Christopher W. Kuzawa[a,g], Dan T.A. Eisenberg[j,k,1*],

[a]Department of Anthropology, Northwestern University, Evanston, IL 60208; [b]Division of Endocrinology, Metabolism and Molecular Medicine, Department of Medicine, Northwestern University, Feinberg School of Medicine, Chicago, IL 60611; [c]Division of Endocrinology, Metabolism and Molecular Medicine, Department of Medicine, Northwestern University Feinberg School of Medicine, Chicago, IL 60611; [d]Center for Genetic Medicine, Northwestern University Feinberg School of Medicine, Chicago, IL 60611; [e]Office of Population Studies Foundation Inc., Cebu City, Philippines; [f]Department of Anthropology, Sociology, and History, University of San Carlos, Cebu City, Philippines; [g]Institute for Policy Research, Northwestern University, Evanston, IL 60208; [h]Child and Brain Development Program, Canadian Institute for Advanced Research, Toronto, ON, Canada, M5G 1Z8; [i]BC Children's Hospital Research Institute, University of British Columbia, Vancouver, BC, Canada,V5Z 4H4; [j]Department of Anthropology, University of Washington, Seattle, WA 98195; [k]Center for Studies in Demography and Ecology, University of Washington, Seattle, WA 98195;

Supplementary Notes:

Comparison of TL across ethnicities. qPCR measured TL does not permit comparisons across populations, but 190 samples from the population for which the samples from this paper come from have had southern blot TL measured[1]. The average age of this sample is 35.96 (± 15.83, range 21.02-68.33), with an average TL of 7.86 kb (± 0.76), and an estimated age related decline in TL of 21.98 bp/year (95% CI 15.81-28.15). This age related decline is non-significantly less than that found in other populations (African=27.7 bp/year; African Americans = 25.6 bp/year; Europeans = 27.3 bp/year)[2]. These three populations from Hansen et al. (2016) have an average age of 43.25. Adjusting the Cebu population TL for the 7.29 years younger they are by assuming the observed 21.98 bp/year attrition rates yields an interpolated TL of 7.70 kb if the mean age was 43.25, the same age as the samples in Hansen et al. (2016). This suggests that the TL in Cebu is longer than that of European and African Americans, but slightly shorter than that of Africans.

**Ethnicity (N)**	**% Female**	**Age (years)**	**LTL (kb)**
Europeans (90)*	63.3	43.9 (18-78)	7.21 (5.39-9.42)
African Americans (97)*	67.1	42.9 (21-79)	7.45 (5.55-9.16)
Africans (100)*	68.0	43.0 (18-79)	7.85 (5.64-10.13)
Cebu, Philippines (190)	50.3	43.3~	7.70 (6.10-9.21)

* from Hansen MEB, et al. (2016) Shorter telomere length in Europeans than in Africans due to polygenetic adaptation. *Human Molecular Genetics*.
†from Eisenberg DT, Kuzawa CW, Hayes MG (2015) Improving qPCR telomere length assays: Controlling for well position effects increases statistical power. *Am J Hum Biol* 27(4):570–5.
~ interpolated to match mean age of other populations - see above text

Calculations for the effect of parity on telomere aging (in years):

Each additional pregnancy was associated with a reduction in TL of 0.016 units (Table 2, Model 3), or an interpolated -50.6 bp/pregnancy based on previous southern blot measured TL in a subset of these samples[2]. The age related decline in TL in 36-69 year old women in this population was previously found to be 0.0043 relative TL units/year (n=1,845, p=7.19 x 10^{-16})[3] or 13.6 bp/year, while the age related decline in TL within the younger and narrow age range in this sample is estimated between -0.027 and -0.047/year (Table 2, Models 1-4). In other populations the age-related decline in mid-late adulthood is approximately 25 bp/year[2]. This implies that one pregnancy is equivalent to between 0.34 and 3.72 years of telomere aging depending on the comparison population. Consistent with this calculation, each additional pregnancy was associated with 0.44-year (0.27 to 0.61 years) increase in DNAmAge acceleration (Table 2, Model 7).

Table S1: Full model estimates for the effect of parity on telomere length (1-4) and DNAmAge (5-8) among young women in the Philippines.

	Telomere Length				DNAmAge			
	(1)	(2)	(3)	(4)	(5)	(6)	(7)	(8)
**Age**	−0.047	−0.029	−0.028	−0.029	0.485	0.667	0.656	0.645
	$p = 0.003^{**}$	$p = 0.071^{+}$	$p = 0.073^{+}$	$p = 0.068^{+}$	$p = 0.293$	$p = 0.157$	$p = 0.158$	$p = 0.165$
**No.Pregnancies**	−0.014	−0.013	−0.014	−0.016	0.363	0.326	0.459	0.510
	$p = 0.025^{*}$	$p = 0.039^{*}$	$p = 0.031^{*}$	$p = 0.020^{*}$	$p = 0.026^{*}$	$p = 0.049^{*}$	$p = 0.007^{**}$	$p = 0.005^{**}$
**PC1**		−0.419	−0.418	−0.419		−11.623	−11.250	−11.219
		$p = 0.065^{+}$	$p = 0.066^{+}$	$p = 0.066^{+}$		$p = 0.084^{+}$	$p = 0.090^{+}$	$p = 0.091^{+}$
**PC2**		−0.154	−0.155	−0.143		5.098	4.793	4.341
		$p = 0.501$	$p = 0.499$	$p = 0.535$		$p = 0.458$	$p = 0.480$	$p = 0.523$
**PC3**		0.005	0.001	0.007		8.510	10.074	9.856
		$p = 0.984$	$p = 0.996$	$p = 0.975$		$p = 0.202$	$p = 0.127$	$p = 0.136$
**PC4**		−0.030	−0.026	−0.033		11.991	10.450	10.511
		$p = 0.894$	$p = 0.909$	$p = 0.883$		$p = 0.069^{+}$	$p = 0.109$	$p = 0.107$
**PC5**		−0.235	−0.237	−0.231		−14.123	−14.033	−13.766
		$p = 0.312$	$p = 0.308$	$p = 0.321$		$p = 0.023^{*}$	$p = 0.022^{*}$	$p = 0.025^{*}$
**PC6**		−0.294	−0.301	−0.291		−4.002	−1.683	−2.286
		$p = 0.204$	$p = 0.194$	$p = 0.211$		$p = 0.548$	$p = 0.799$	$p = 0.731$
**PC7**		−0.506	−0.515	−0.517		18.513	21.220	21.471
		$p = 0.031^{*}$	$p = 0.029^{*}$	$p = 0.028^{*}$		$p = 0.008^{**}$	$p = 0.003^{**}$	$p = 0.002^{**}$
**PC8**		0.421	0.421	0.425		3.987	3.930	3.829
		$p = 0.067^{+}$	$p = 0.067^{+}$	$p = 0.064^{+}$		$p = 0.534$	$p = 0.535$	$p = 0.545$
**PC9**		−0.462	−0.460	−0.465		−1.730	−2.332	−2.337
		$p = 0.040^{*}$	$p = 0.041^{*}$	$p = 0.039^{*}$		$p = 0.789$	$p = 0.715$	$p = 0.714$
**PC10**		0.555	0.551	0.544		−2.701	−1.329	−1.043
		$p = 0.021^{*}$	$p = 0.022^{*}$	$p = 0.023^{*}$		$p = 0.690$	$p = 0.843$	$p = 0.876$
**SES-score**		−0.006	−0.006	−0.004		−0.180	−0.214	−0.291
		$p = 0.143$	$p = 0.161$	$p = 0.395$		$p = 0.146$	$p = 0.081^{+}$	$p = 0.055^{+}$
**Urbanicity-score**		0.002	0.002	0.002		0.015	0.017	0.017
		$p < 0.001^{**}$	$p < 0.001^{**}$	$p < 0.001^{**}$		$p = 0.270$	$p = 0.213$	$p = 0.226$
**Currently Pregnancy (Y)**			0.011	0.011			−1.472	−1.460
			$p = 0.534$	$p = 0.540$			$p = 0.001^{**}$	$p = 0.001^{**}$
**No.Pregnancies*SES-score**				−0.004				0.106
				$p = 0.362$				$p = 0.385$
**Intercept**	1.826	1.337	1.332	1.343	14.818	10.319	10.611	10.850
	$p < 0.001^{**}$	$p < 0.001^{**}$	$p < 0.001^{**}$	$p < 0.001^{**}$	$p = 0.138$	$p = 0.318$	$p = 0.297$	$p = 0.287$
Observations	821	821	821	821	397	397	397	397
R^2	0.018	0.079	0.079	0.080	0.016	0.075	0.103	0.104
Adjusted R^2	0.015	0.063	0.062	0.062	0.011	0.041	0.067	0.067
Residual Std. Error	0.161 (df = 818)	0.157 (df = 806)	0.157 (df = 805)	0.157 (df = 804)	3.165 (df = 394)	3.117 (df = 382)	3.074 (df = 381)	3.075 (df = 380)
F Statistic	7.347^{**} (df = 2; 818)	4.929^{**} (df = 14; 806)	4.623^{**} (df = 15; 805)	4.385^{**} (df = 16; 804)	3.220^{*} (df = 2; 394)	2.214^{**} (df = 14; 382)	2.902^{**} (df = 15; 381)	2.766^{**} (df = 16; 380)

Note: +p<0.1; *p<0.05; **p<0.01; ***p<0.001

References

1. Eisenberg DT, Kuzawa CW, Hayes MG. Improving qPCR telomere length assays: Controlling for well position effects increases statistical power. Am J Hum Biol, 2015, 27:570–5
2. Hansen MEB, Hunt SC, Stone RC, Horvath K, Herbig U, Ranciaro A, Hirbo J, Beggs W, Reiner AP, Wilson JG, Kimura M, De Vivo I, Chen MM, Kark JD, Levy D, Nyambo T, Tishkoff SA, Aviv A. Shorter telomere length in Europeans than in Africans due to polygenetic adaptation. Hum Mol Genet, 2016
3. Eisenberg DT, Hayes MG, Kuzawa CW. Delayed paternal age of reproduction in humans is associated with longer telomeres across two generations of descendants. Proc Natl Acad Sci, 2012, 109:10251–6
